# Supplementary material for: The interactome of intact mitochondria by cross-linking mass spectrometry provides evidence for coexisting respiratory supercomplexes
Source: Mol Cell Proteomics. 2017 Dec 8;17(2):216–32. doi: 10.1074/mcp.RA117.000470 (PMC5795388; doi:10.1074/mcp.RA117.000470)
Supplement: Supplemental Data [file supp_RA117.000470_133922_0_supp_23978_fzffwf.zip › spectra_annotation/mito_DR_spectra_annotation/353-1-1-1-7-1.pdf]

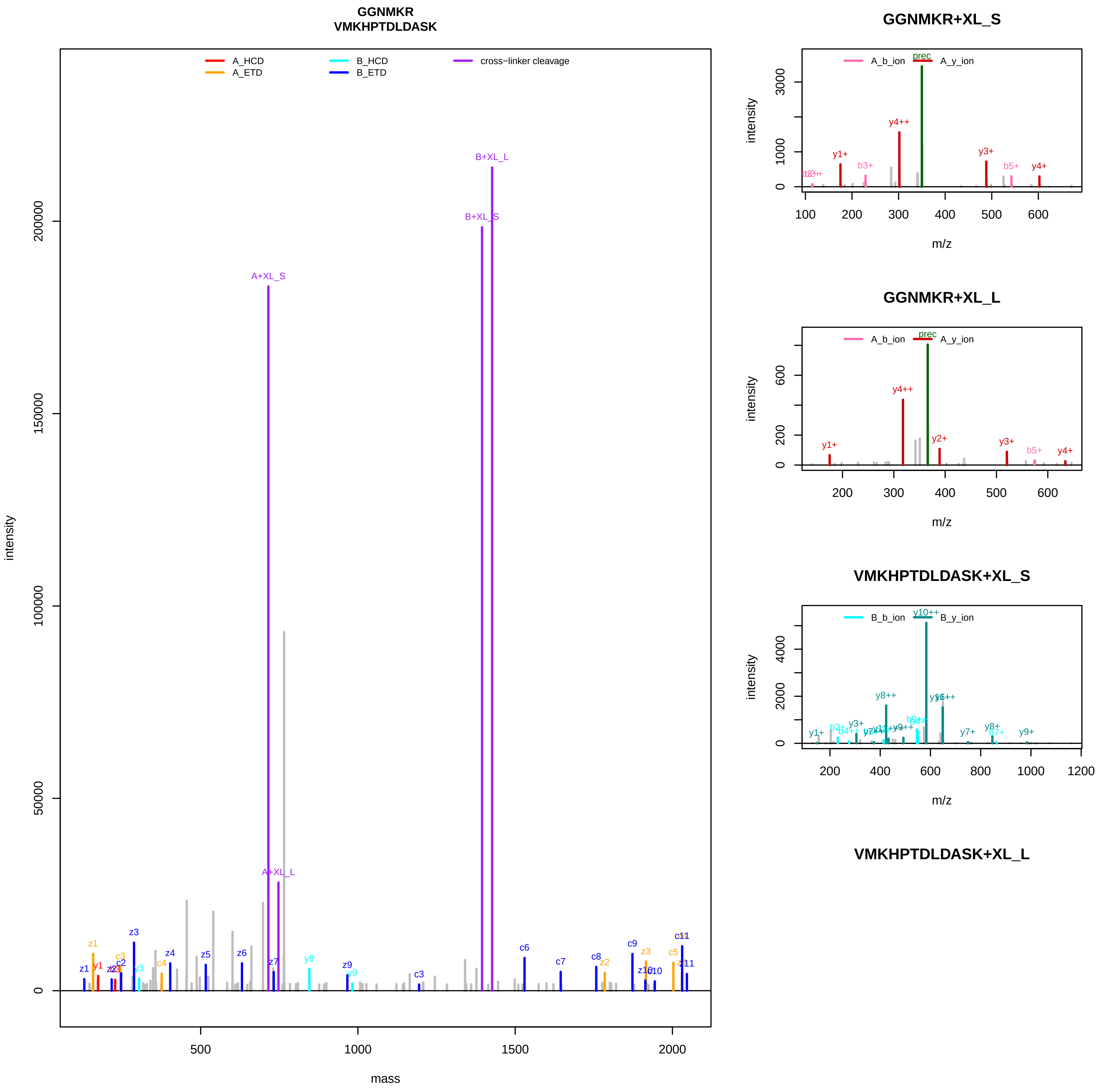

GGNMKR
VMKHPTDLDASK
A_HCD
A_ETD
B_HCD
B_ETD
cross−linker cleavage
B+XL_L
B+XL_S
A+XL_S
A+XL_L
intensity
mass
0
50000
100000
150000
200000
500
1000
1500
2000
z1
y1
c2
z3
z4
c4
z5
z6
z7
y8
z9
y9
c3
c6
c7
c8
z2
c9
z3
c5
z11
GGNMKR+XL_S
A_b_ion
A_y_ion
prec
y4++
y1+
b3+
y3+
b5+
y4+
intensity
m/z
GGNMKR+XL_L
A_b_ion
A_y_ion
prec
y4++
y1+
y2+
y3+
b5+
y4+
intensity
m/z
VMKHPTDLDASK+XL_S
B_b_ion
B_y_ion
y10++
y8++
y1+
y3+
y7+
y8+
y9+
b7+
intensity
m/z
VMKHPTDLDASK+XL_L
